# A comparison of risk factors for mortality from heart failure in Asian and non-Asian populations: An overview of individual participant data from 32 prospective cohorts from the Asia-Pacific Region

**DOI:** 10.1186/1471-2261-14-61

**Published:** 2014-05-03

**Authors:** Rachel R Huxley, Federica Barzi, Jean Woo, Graham Giles, Tai Hing Lam, Kazem Rahimi, Suma Konety, Takayoshi Ohkubo, Sun Ha Jee, Xianghua Fang, Mark Woodward

**Affiliations:** 1 Epidemiology & Biostatistics Division, School of Population Health, University of Queensland, Brisbane, Australia; 2 The George Institute for Global Health, University of Sydney, Sydney, Australia; 3 Department of Medicine and Therapeutics, Faculty of Medicine, The Chinese University of Hong Kong, Hong Kong, China; 4 Cancer Epidemiology Centre, The Cancer Council Victoria, Melbourne, Australia; 5 School of Public Health/Department of Community Medicine, The University of Hong Kong, Hong Kong, China; 6 George Centre for Healthcare Innovation, University of Oxford, Oxford, UK; 7 Department of Cardiovascular Medicine, University of Oxford, Oxford, UK; 8 Division of Epidemiology and Community Health, School of Public Health, University of Minnesota, Minneapolis, Minnesota, USA; 9 Department of Health Science, Shiga University of Medical Science, Shiga, Japan; 10 Institute for Health Promotion, Graduate School of Public Health, Yonsei University, Seoul, Korea; 11 Department of Epidemiology and Social Medicine, Xuanwu Hospital, Capital Medical University, Beijing, China; 12 Division of Epidemiology, Johns Hopkins University, Baltimore, USA

**Keywords:** Meta-analysis, Heart failure, Epidemiology

## Abstract

**Background:**

Most of what is known regarding the epidemiology of mortality from heart failure (HF) comes from studies within Western populations with few data available from the Asia-Pacific region where the burden of heart failure is increasing.

**Methods:**

Individual level data from 543694 (85% Asian; 36% female) participants from 32 cohorts in the Asia Pacific Cohort Studies Collaboration were included in the analysis. Adjusted hazard ratios (HR) and 95% confidence intervals (CI) for mortality from HF were estimated separately for Asians and non-Asians for a quintet of cardiovascular risk factors: systolic blood pressure, diabetes, body mass index, cigarette smoking and total cholesterol. All analyses were stratified by sex and study.

**Results:**

During 3,793,229 person years of follow-up there were 614 HF deaths (80% Asian). The positive associations between elevated blood pressure, obesity, and cigarette smoking were consistent for Asians and non-Asians. There was evidence to indicate that diabetes was a weaker risk factor for death from HF for Asians compared with non-Asians: HR 1.26 (95% CI: 0.74-2.13) versus 3.04 (95% CI 1.76-5.25) respectively; p for interaction = 0.022. Additional adjustment for covariates did not materially change the overall associations. There was no good evidence to indicate that total cholesterol was a risk factor for HF mortality in either population.

**Conclusions:**

Most traditional cardiovascular risk factors including elevated blood pressure, obesity and cigarette smoking appear to operate similarly to increase the risk of death from HF in Asians and non-Asians populations alike.

## Background

Heart failure (HF) is a complex syndrome with a multifactorial aetiology and is a major cause of death and disability in higher-income countries where it is estimated that 2% of adults are affected [1]. The incidence of HF is strongly age-related so that the prevalence of the condition rises to 6-10% in those over 65 years of age [1]. In the United States (US) alone, an estimated 550,000 new cases occur annually [2] and in 2008, 1 in 9 death certificates mentioned heart failure [3]. Globally, it has been estimated that 23 million individuals worldwide have HF [4] although this may be an underestimate given the lack of reliable population-based studies of the prevalence and incidence of HF for Asia and elsewhere [5,6]. What data there are from Asia suggest that hospitalization for HF is increasing in the region [7,8].

Our knowledge regarding the causes of HF has chiefly been derived from studies from the US [9] and Europe [10] with relatively few data from less developed parts of the world including Asia [11]. As with other forms of cardiovascular disease, the major lifestyle risk factors for HF are reported to include elevated blood pressure, obesity, smoking, and diabetes [12]. Whether these risk factors exert the same effect on Asian populations that tend to have markedly different risk factor profiles compared with non-Asians [13] remains to be determined. In this paper we report on the associations between traditional cardiovascular risk factors and mortality from HF in the Asia-Pacific region and contextualize these findings with respect to the current literature using data from the Asia Pacific Cohort Studies Collaboration (APCSC) [14,15].

## Methods

### Ethics approval

All of the individual studies that participated in APCSC obtained ethics approval from their own ethics approval boards. All participants were aged 18 or over and provided informed consent in accordance with the principles of the Declaration of Helsinki.

Details of the APCSC have been described elsewhere [14,15]. In brief, a study was eligible for inclusion if the population was drawn from the Asia Pacific region; it had a prospective cohort study design without selection being consequent to pre-existing disease; it had accumulated at least 5000 person-years of follow-up; date of birth (or age), sex and blood pressure were recorded at baseline; and date or age at death was recorded during follow-up. Cohorts were excluded if they were based on a positive disease history or diagnosis. Cohorts were classified as Asian if the participants were recruited from mainland China, Hong Kong, South Korea, Japan or Taiwan, and Australian if the cohorts were recruited within Australia. The majority of cohorts were population-based but several of the cohorts were sourced from occupational settings (e.g. KMIC, Guangzhou Occupational, Beijing Steelworkers, and Civil Service Workers).

In most studies, blood pressure was measured at rest in the seated position using a standard mercury sphygmomanometer [16]. TC was obtained using previously described methods [17]. Body mass index (BMI) was calculated as weight (kg) divided by squared height (m^2^) [18]. The diabetes status of individual participants was determined on the basis of a self-reported history of diabetes at baseline or by applying the World Health Organization (WHO) criteria to baseline blood glucose levels [19]. Cigarette smoking was classified based on self-report at baseline as ‘ever’ versus ‘never’ smoking [20].

### End-point

Most studies used database linkage to identify deaths, while others also included scheduled follow-up visits or examined hospital records Information on fatal events was classified according to the Ninth Revision of the International Classification of Diseases (ICD-9). The end point considered in this analysis was fatal HF (ICD-9 428). Data quality of the individual cohorts was centrally checked and, if needed, data were recoded for comparability across studies. Where necessary, further details were sought from the principal investigators of each individual study.

### Statistical analyses

All analyses used individual participant data, restricted to participants aged ≥20 years at the time of the baseline survey. Cox proportional hazards regression was used to estimate hazard ratios (HRs) and 95% confidence intervals (95% CI) for HF for each risk factor, after adjustment for age. All analyses were stratified by sex and study. For linear associations HRs for a unit increase were derived. BMI and HF exhibited a non-linear relationship so the HRs across quarters were reported. In addition, the HR’s across five sequential categories of BMI were reported: <18.5, 18.5–21.9, 22–24.9, 25–29.9 and ≥30 kg/m^2^[21]. The 95% CI for categories of BMI were obtained by the method of floating absolute risks [22]. The associations for continuous variables were adjusted for regression dilution bias using repeated measurement data [23]. The analyses were repeated within subgroups according to sex, age (≤75 v > 75 years), and region (Asia v Australia) and a p-value less than 0.05 was considered evidence of an interaction. In a sensitivity analysis, the impact of adjustment for SBP, smoking, BMI and diabetes was explored using a restricted sample that had information on these variables. In a further sensitivity analysis, the data were left-censored to exclude events that occurred during the first two years of follow-up. We also examined whether there was an interaction with history of cardiovascular disease at study baseline (CVD) by including an interaction term in the model. Data provided to the Secretariat were checked for completeness and consistency and recoded, when necessary, to maximize comparability across cohorts. Summary reports were referred back to principal investigators of each collaborating study for review and confirmation. Analyses were performed using SAS, version 9.2 and Stata version 11.

## Results

A total of 543694 (85% Asian; 36% female) participants from 32 cohorts were included in this analysis (Table 1). Due to missing values for some variables, the number of subjects available for analysis varied according each to risk factor.

**Table 1:** Baseline characteristics of participating studies in Australia and Asia in the Asia Pacific Cohort Studies Collaboration

**Study name (Country)**	**N**	**Baseline years**	**Female (%)**	**Age (yrs)**	**HF deaths**	**FU (yrs)**	**SBP (mmHg)**	**BMI (kg/m2)**	**TC (mmol/L)**	**Diab (%)**	**Smoke (%)**
ALSA (Aus)	1613	1992-93	48	78	20	5	148	26.0	5.8	8.3	7.7
ANHF (Aus)	9277	1989-90	51	43	3	8	126	25.4	5.5	1.9	24.1
Busselton (Aus)	7866	1966-81	52	45	50	27	138	24.6	5.9	3.5	33.8
Canberra (Aus)	833	1990-91	46	77	16	9	145	-	-	6.8	11.3
Melbourne (Aus)	41286	1990-94	59	55	18	9	138	26.9	5.5	5.4	11.3
Perth (Aus)	10230	1978-94	48	45	5	14	130	25.2	5.8	2.1	25.5
WAAAA Screenees (Aus)	12203	1996-99	0	72	10	3	157	26.9	-	11.6	10.9
*Total Australia*	*83308*		*47*	*55*	*122*	*8*	*139*	*26.3*	*5.6*	*5.4*	*16.4*
Aito Town (Japan)	1717	1980-83	57	51	9	15	136	22.6	4.6	2.7	28.6
Akabane (Japan)	1836	1985-86	56	54	16	11	125	22.5	5.0	2.5	28.0
Anzhen (China)	8378	1991	55	54	3	4	129	23.9	-	0.0	28.5
Beijing Steelworkers (China)	8957	1970	12	36	15	28	123	-	-	-	-
Civil Service Workers (Japan)	9319	1990-92	33	47	1	7	126	22.5	5.2	1.8	37.9
CVDFACTS (Taiwan)	5730	1988-96	55	47	4	6	118	23.5	5.0	2.7	22.2
East Beijing (China)	1128	1977-94	51	44	3	17	125	23.6	-	5.6	28.6
Fangshan (China)	2625	1991-92	67	47	1	4	136	24.4	4.6	7.1	39.3
Guangzhou Occupational (China)	167377	1985-97	22	42	11	7	115	22.6	5.3	10.5	47.6
Hisayama (Japan)	1616	1961	56	56	24	25	135	21.6	4.1	0.0	42.7
Hong Kong	3006	1985-91	58	79	4	3	150	21.9	5.3	8.6	18.6
Huashan (China)	1868	1990-92	52	53	2	3	126	23.3	4.6	13.7	24.9
Kinmen (Taiwan)	2793	1993-96	48	63	90	3	137	23.4	-	8.6	8.3
KMIC (Korea)	183600	1992	37	44	7	4	122	23.0	5.0	7.7	38.5
Konan (Japan)	1226	1987-95	55	52	9	6	130	21.9	4.9	12.6	30.1
Miyama (Japan)	1078	1988-90	56	61	11	7	132	22.1	5.1	0.0	29.5
Ohasama (Japan)	2240	1992-93	64	60	4	4	128	23.3	5.0	10.9	20.0
Saitama (Japan)	3624	1986-90	62	55	28	11	135	22.4	5.0	1.7	28.4
Seven Cities Cohorts (China)	10811	1987	55	54	139	3	130	22.6	5.0	1.2	35.1
Shibata (Japan)	2350	1977	58	57	34	20	131	22.4	4.6	1.1	33.1
Shigaraki Town (Japan)	3758	1991-97	60	57	7	4	132	22.5	5.0	7.2	28.8
Shirakawa (Japan)	4643	1974-79	54	48	27	18	127	21.5	4.6	0.9	34.9
Six Cohorts (China)	19387	1982-86	47	45	6	9	119	21.2	4.2	0.0	46.0
Tanno/Soubetsu (Japan)	1984	1977	53	51	16	16	133	23.6	4.9	7.2	38.6
Tianjin (China)	9335	1984	51	55	21	6	136	23.5	-	0.0	50.9
*Total Asia*	*460386*		*34*	*45*	*492*	*6*	*121*	*22.8*	*4.9*	*6.9*	*41.4*
Total	543694		36	46	614	7	124	23.6	5.1	6.5	37.3

FU = mean follow-up in years; SBP = systolic blood pressure; TC = total cholesterol; diab = diabetes; HF = death from heart failure; NHS = National Health Survey; ALSA = Australian Longitudinal Study of Aging; ANHF = Australian National Heart Foundation. WAAAA = West Australian Abdominal Aortic Aneurysm; CISCH = Capital Iron and Steel Hospital; KMIC = Korean Medical Insurance Corporation. - = no data available.

### Systolic blood pressure

**Figure 1:**
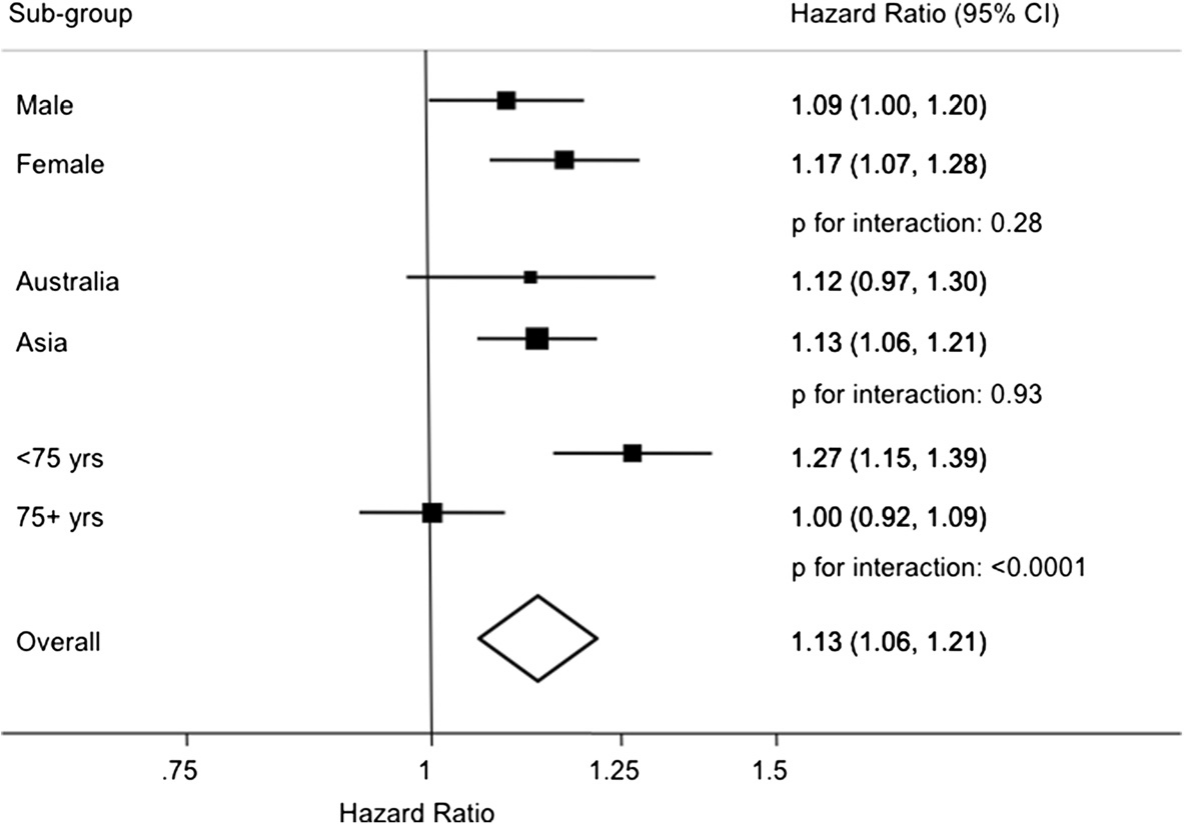
**Hazard ratios for fatal heart failure associated with a 10 mmHg increment in systolic blood pressure, adjusted for age and regression dilution bias and stratified by sex and study in the Asia Pacific Cohort Studies Collaboration, both by subgroup (male versus female; Asia versus Australia; ≤ 75 yrs vs > 75 yrs) and overall.** Bars show 95% confidence intervals. The vertical dimension of the diamond indicates the overall estimate and the horizontal dimension indicates the 95% confidence interval.

Overall, 3,793,229 person years of follow-up data contributed to the analysis of SBP with HF during which time 614 deaths (80% Asian) from HF were recorded. In the Asian cohorts, 56% of HF deaths were from Japan, 42% from China and the remainder from Hong Kong, Taiwan, Japan and Korea. There was a significant and linear positive association between SBP and the risk of mortality from HF (p = 0.0003). Every 10 mmHg increment in SBP increased the risk by 13% (95% CI: 6-21%) with no evidence that the effect varied by sex (p = 0.28) or region (p = 0.93, Figure 1). There was a highly significant interaction with age (p < 0.0001), such that the association was only observed for younger compared with older individuals: HR 1.27 (1.15-1.39) and HR 1.00 (0.92-1.09) in individuals ≤75 years and >75 years, respectively. Adjusting for smoking, BMI and diabetes did not materially change the associations (Additional file 1: WebFigure 1). There was some evidence that the association differed by whether or not the individual had a history of cardiovascular disease (CVD) at study baseline (Additional file 1: WebTable 1); In those individuals with no CVD history, there was a positive association whereas there was no apparent association between SBP and heart failure mortality in those with a history of CVD (p _interaction with history of CVD_ = 0.024). This may have been a chance finding due to the small number (<100) of events that occurred in those with a history of CVD.

### Body mass index

**Figure 2:**
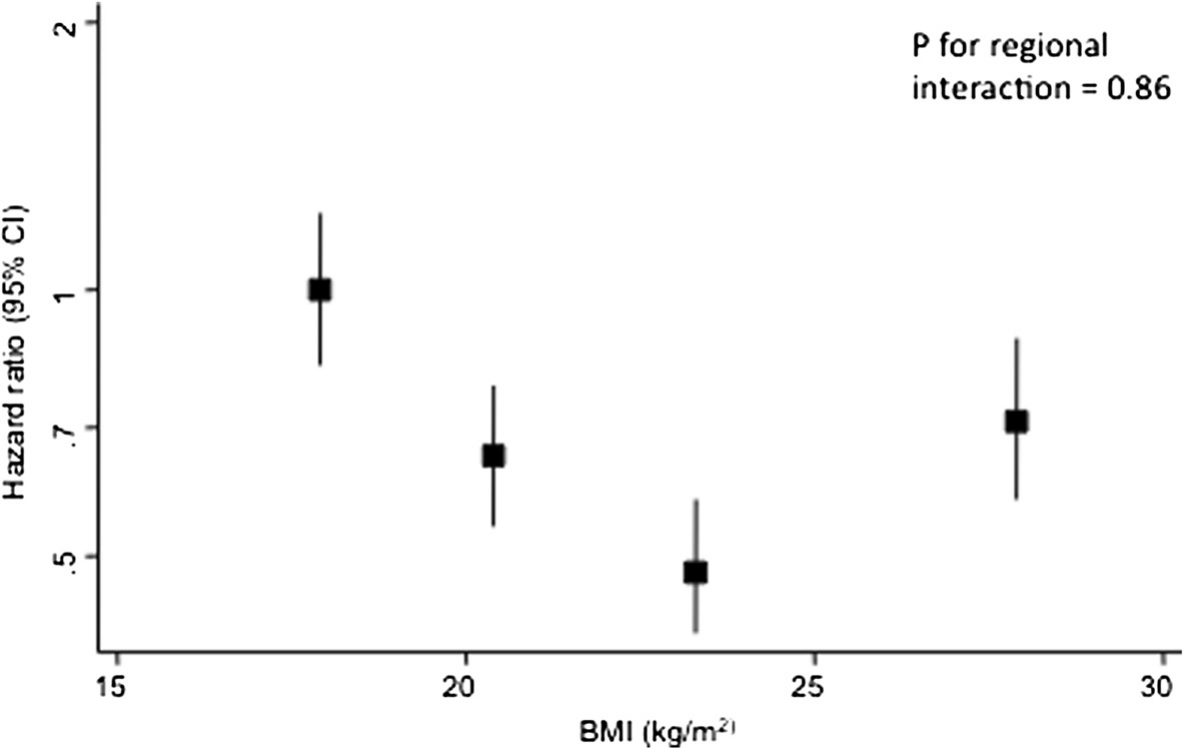
**Hazard ratios for fatal heart failure by fourths of body mass index adjusted for age and regression dilution; stratified by sex and study.** Bars show 95% confidence intervals. Baseline fourths were ≤21.1, 21.2-23.2, 23.3-25.4, ≥25.5 kg/m^2^.

The age-adjusted analysis between BMI and HF mortality was based on a total of 2,430,378 person-years and 494 events. There was a U-shaped relationship across quarters of BMI (Figure 2), with the second and third quarters having the lowest risk of mortality compared with the first quarter. There was no indication that the association differed by region (p = 0.86), sex (p = 0.65), or age (p = 0.95). When the relationship was examined using the pre-specified categories for weight with normal weight (18.5–21.9 kg/m^2^) as the reference group the U-shaped relation was more evident (Table 2). Compared with the reference group, both underweight and obese individuals had more than a 60% increased risk of dying from HF compared with normal weight individuals: HR 1.68 (95% CI: 1.34-2.11) and 1.69 (1.17-2.43) for underweight and obese individuals, respectively which remained unchanged after adjustment for cigarette smoking or after two years left censoring (Table 2). The relationship was consistent in those with and without a history of CVD at study baseline (p _interaction with history of CVD_ = 0.92; Additional file 1: WebTable 1).

**Table 2:** The categorical association between body mass index (BMI) and mortality from heart failure in the Asia Pacific Cohort Studies Collaboration

**BMI category (kg/m**^**2**^**)**	**Age, sex and study adjusted**		**Age, sex, study, smoking adjusted**		**2-year left censored**	
	**HF deaths**	**HR (95% CI)**	**HF deaths**	**HR (95% CI)**	**HF deaths**	**HR (95% CI)**
< 18.5 (underweight)	100	1.68 (1.34-2.11)	100	1.72 (1.37-2.16)	79	1.78 (1.37-2.30)
18.5 – 21.9 (normal, ref)	157	1.00 (0.84-1.18)	151	1.00 (0.84-1.19)	125	1.00 (0.83-1.21)
22 – 24.9	103	0.81 (0.67-0.98)	101	0.83 (0.68-1.01)	87	0.86 (0.69-1.05)
25 – 29.9 (overweight)	91	0.98 (0.80-1.22)	90	1.02 (0.83-1.27)	76	1.04 (0.82-1.31)
> 30 (obese)	33	1.69 (1.17-2.43)	33	1.76 (1.22-2.54)	27	1.75 (1.16-2.63)

### Lifetime cigarette smoking

**Figure 3:**
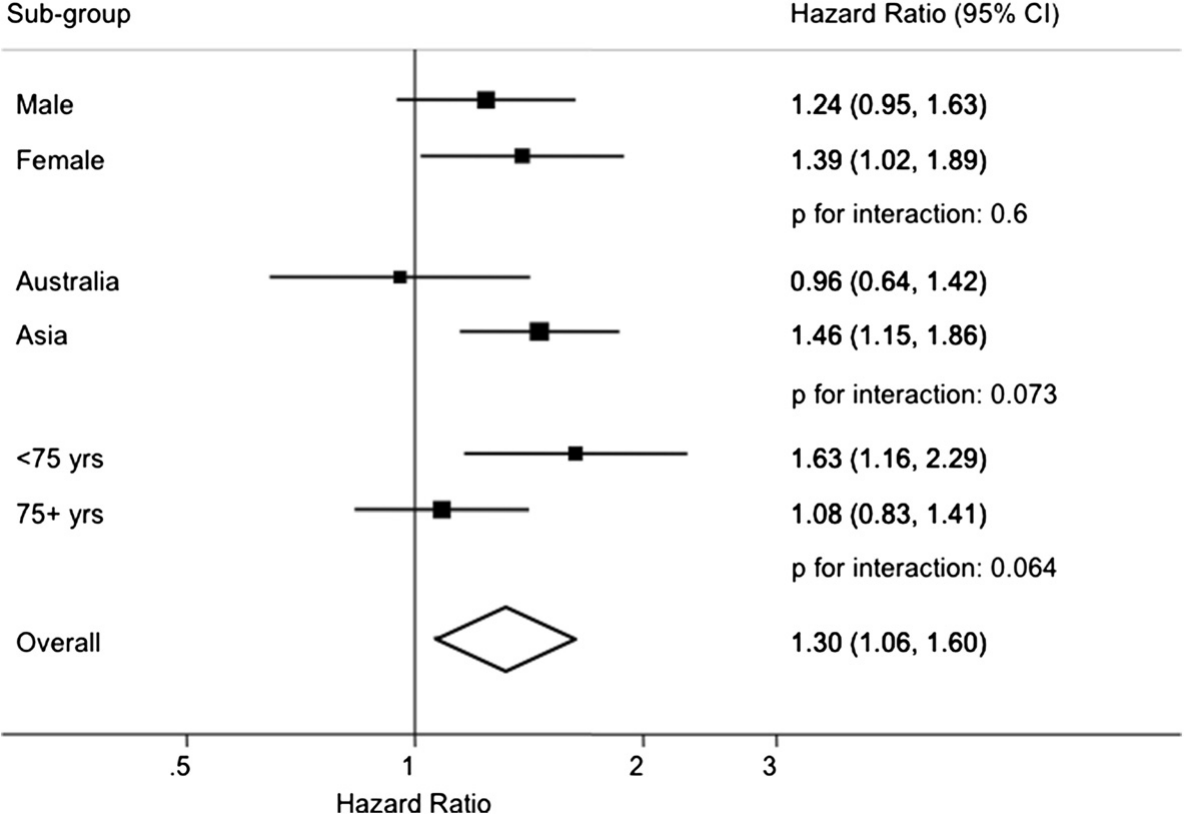
**Hazard ratios for fatal heart failure associated with cigarette smoking (ever versus never) adjusted for age and stratified by sex and study in the Asia Pacific Cohort Studies Collaboration.** Conventions as in Figure 1.

A total of 3,443,811 person years and 579 events contributed to this analysis. Compared with never smokers ever smoking was significantly associated with 30% increased risk of mortality (95% CI: 6-60%; Figure 3). There was no evidence that the association differed by region (p = 0.073), sex (p = 0.60) or age (p = 0.064). The results remained largely unchanged after adjusting for SBP and BMI (Additional file 1: WebFigure 2). The relationship was consistent in those with and without a history of CVD at study baseline (p _interaction with history of CVD_ = 0.10; Additional file 1: WebTable 1).

### Diabetes

**Figure 4:**
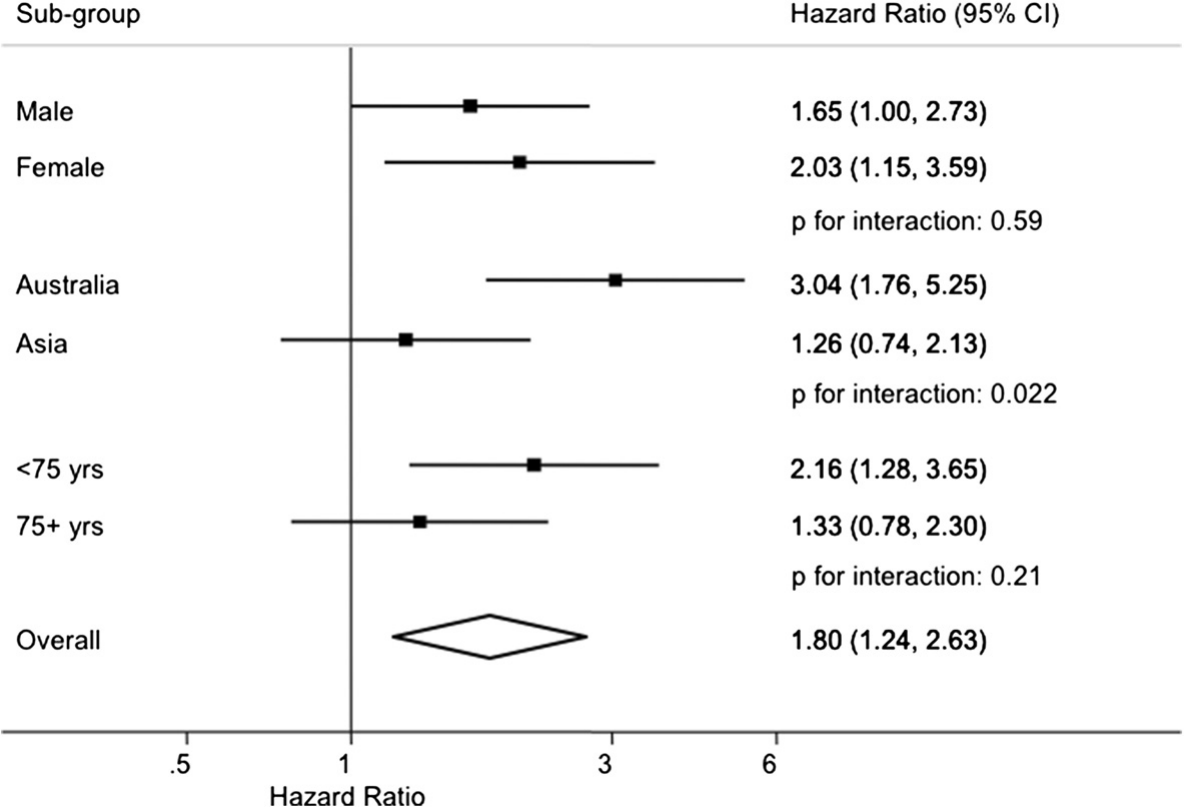
**Hazard ratios for fatal heart failure associated with diabetes (yes versus no) adjusted for age and regression dilution bias and stratified by sex and study in the Asia Pacific Cohort Studies Collaboration.** Conventions as in Figure 1.

Based on 1,996,642 person years of data and 496 events, individuals with diabetes had 80% (95% CI: 1.24-2.63) greater risk of death from HF compared with those without the condition, with no evidence to suggest that the relationship differed significantly by sex (p = 0.59) or by age (p = 0.21) (Figure 4). There was suggestive evidence of a stronger effect in cohorts from Australia compared with Asia: HR 3.04 (1.76-5.25) versus 1.26 (0.74-2.13) (p = 0.022). There was no material change in these associations once the effect of other covariates was considered (Additional file 1: WebFigure 3). The relationship was consistent in those with and without a history of CVD at study baseline (p _interaction with history of CVD_ = 0.15; Additional file 1: WebTable 1).

### Total cholesterol

**Figure 5:**
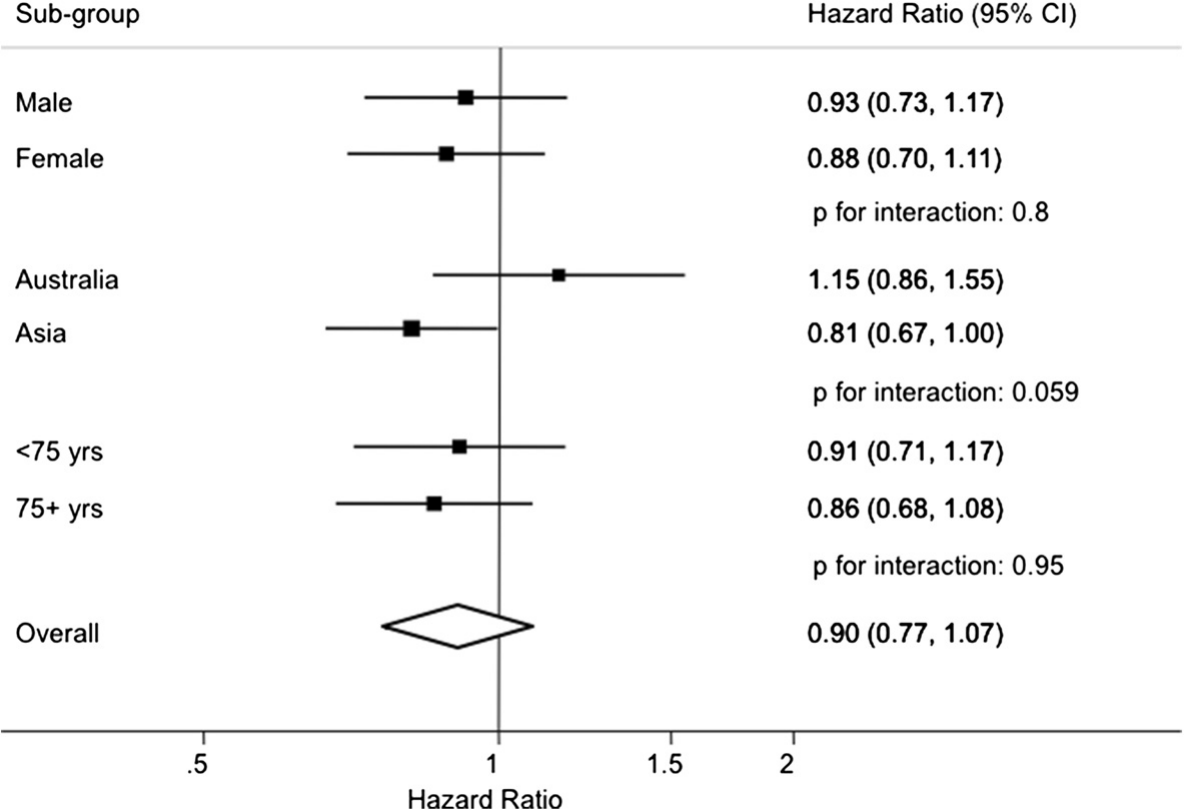
**Hazard ratios for fatal heart failure associated with a 1 mmol/L increment in total cholesterol adjusted for age and regression dilution bias and stratified by sex and study in the Asia Pacific Cohort Studies Collaboration.** Conventions as in Figure 1.

Information on the association between TC and HF was based on 2,145,819 person years and 365 events. Test for linearity indicated that there was a weak inverse association between TC and HF (p = 0.01). A 1 mmol/L increment in TC was associated with a non-significant reduction in HF mortality with no significant evidence of a regional interaction: HR 0.90 (95% CI: 0.77-1.07); p = 0.059; Figure 5. The HR remained largely unchanged after additional adjustment for cigarette smoking, SBP and BMI: HR 0.93 (0.77-1.11) per 1 mmol/L increase in TC (Additional file 1: WebFigure 4). There was also no material effect after excluding the first two years of follow up (HR 0.98 [95% CI 0.81-1.17]). The relationship was consistent in those with and without a history of CVD at study baseline (p _interaction with history of CVD_ = 0.45; Additional file 1: WebTable 1).

## Discussion

This study comprises the largest amount of prospective data to date on the relationship between major cardiovascular risk factors with HF mortality in populations from the Asia-Pacific region. The key findings from this individual participant data meta-analysis indicate positive and independent relationships between elevated blood pressure, obesity, diabetes and cigarette smoking with death from HF in Asians and non-Asians, but no evidence of an association with TC. With the possible exception for blood pressure, the relationships were consistent in those with and without a history of CVD at study baseline. Overall, these current findings are broadly consistent with those reported from Western population-based studies, including Framingham [24,25] and NHANES [12]. Both of these US studies reported hypertension to be a major, if not the leading, modifiable cause of incident HF in addition to diabetes, smoking and obesity [24,25]. Comparative population-based data from Asia are sparse, but those derived from hospital-based studies have also indicated the frequently high co-occurrence of hypertension and diabetes among individuals hospitalized for HF [26].

That diabetes may also be an independent risk factor for incident HF was first shown by the Framingham study where a clinical history of diabetes was associated with a two-fold increased risk in men and a five-fold increased risk for women [25]. Since then, numerous studies have reported higher incidence rates of HF among those with diabetes compared with those without the condition, as well as a relatively higher relative risk of HF in populations of women and young people [27,28]. Data from the current study, confirmed an independent association between diabetes and mortality from HF for non-Asians and (to a lesser extent) Asians. That diabetes may have a slightly weaker association with mortality from HF in Asians than in non-Asians is an intriguing finding that warrants further investigation.

In the current study, a U-shaped association between BMI and mortality from HF was observed such that individuals who were either underweight or obese were at similarly greater risk of dying from HF compared with those of normal weight. This remained unaffected by adjustment for smoking, which may have operated as a confounder of the association given that smokers tend to be relatively leaner than their non-smoking counterparts. Efforts to reduce the impact of reverse causality by excluding the first two years of follow-up also had little impact on the relationship. Our observation that underweight is a risk factor for mortality from HF is tangentially supported by previous hospital-based studies that have shown patients with chronic HF who are underweight have a lower survival than those of normal weight or who are overweight or obese [29]. The increased risk of HF death for underweight individuals may be due to cardiac cachexia, a wasting syndrome observed in patients with advanced HF that has no accepted definition but is characterized by significant weight loss in the absence of peripheral edema [30,31]. Studies have demonstrated that many patients with advanced HF are malnourished, with a calorie and protein intake that is inadequate to meet their energy requirements [32,33]. But, this is unlikely to account for all of the increased risk as even in the absence of cachexia studies have shown that the increased risk of HF for the underweight individual remains [34]. The relationship between low BMI with increased risk of mortality from heart failure may also reflect pre-existing heart failure at study baseline. We did not have information on prevalent HF so could not examine this further but in the sensitivity analysis comparing those with and without a history of CVD at study baseline, the relationship between BMI with mortality from HF was the same. Perhaps of more relevance to Western countries, is the increased risk of mortality from HF for obese compared with relatively lean individuals that we, and other authors, have shown. In Framingham, for example, there was a continuous association between BMI and risk of new onset HF and each unit increase in BMI was associated with a 5% increase in the risk of HF for men and 7% for women [35].

The epidemiological literature describing the relationship between TC and mortality from HF is inconsistent; some studies have reported a positive [36] or inverse relation [37] between TC and mortality from HF, but others including NHANES have shown no association [12]. In the current study there was no good evidence of an association between TC with mortality from HF –either positive or inverse- as shown by the confidence intervals around the point estimate spanning unity in both the unadjusted and adjusted models. This is consistent with randomized data from two clinical trials –CORONA [38] and GISSI-HF [39]–which demonstrated that in patients with HF the incidence of cardiovascular events, which are greatly driven by non-atherosclerotic events, was not importantly affected with statin therapy. Moreover, the Cholesterol Treatment Trialist’s showed that LDL-cholesterol lowering with statin therapy has no benefit on cardiac deaths due to non-occlusive mechanisms, such as HF [40].

There are some important limitations of this analysis. The lack of a universal definition of HF, and between study differences in its diagnosis and reporting, may have introduced bias. For example, misdiagnosis of HF as stroke, myocardial infarction or IHD in the early 1990’s has been suggested to account for the observed increase in IHD mortality between 1990–1995 in Japan [41]. In the current analysis, over half of the data from Asia were derived from Japanese cohorts, many of which were initiated in the early 1990’s. Therefore, it is conceivable that there was some under-reporting of mortality from HF in these cohorts; such misclassification is likely to draw estimates of association towards the null. We also did not have information on incident heart failure which precludes examination of the possible effects of reverse causality on the results (i.e. whether the association between a particular risk factor with mortality from heart failure is impacted by the development on heart failure at some point during follow-up). Another limitation of our data was the lack of information on rheumatic heart disease, coronary artery disease and Chagas’ disease, which are major causes of HF in South Asia and China [8] and which have been reported to be independent predictors of HF risk [42]. Finally, we were unable to examine the relationships between emerging cardiovascular risk factors with HF. In a recent report from the Strong Heart Study, inflammatory markers were shown to be associated with incident HF, although the relationships were substantially attenuated after adjusting for more traditional coronary risk factors [43].

## Conclusions

In summary, findings from this current study indicate that several traditional and modifiable cardiovascular risk factors, namely elevated blood pressure, diabetes, obesity and cigarette smoking, are independently associated with mortality from HF for Asian and non-Asian populations alike in a broadly consistent manner.

## Appendix

APCSC Executive Committee

M. Woodward (Chair), X. Fang, D.F. Gu, R. Huxley, Y. Imai, H.C. Kim, T.H. Lam, W.H. Pan, A. Rodgers, I. Suh, H. Ueshima

Participating Studies and Principal Collaborators in APCSC

Aito Town: A. Okayama, H. Ueshima, H. Maegawa; Akabane: M. Nakamura, N. Aoki; Anzhen02: Z.S. Wu; Anzhen: C.H. Yao, Z.S. Wu; Australian Longitudinal Study of Aging: Mary Luszcz; Australian National Heart Foundation: T.A. Welborn; Beijing Aging: Z. Tang; Beijing Steelworkers: L.S. Liu, J.X. Xie; Blood Donors’ Health: R. Norton, S. Ameratunga, S. MacMahon, G. Whitlock; Busselton: M.W. Knuiman; Canberra-Queanbeyan: H. Christensen; Capital Iron and Steel Company: X.G. Wu; CISCH: J. Zhou, X.H. Yu; Civil Service Workers: A. Tamakoshi; CVDFACTS: W.H. Pan; East Beijing: Z.L. Wu, L.Q. Chen, G.L. Shan; Electricity Generating Authority of Thailand: P. Sritara; Fangshan: D.F. Gu, X.F. Duan; Fletcher Challenge: S. MacMahon, R. Norton, G. Whitlock, R. Jackson; Guangzhou: Y.H. Li; Guangzhou Occupational: T.H. Lam, C.Q. Jiang; Hisayama: Y. Kiyohara, H. Arima, M. Iida; Hong Kong: J. Woo, S.C. Ho; Huashan: Z. Hong, M.S. Huang, B. Zhou; Kinmen: J.L. Fuh; Konan: H. Ueshima, Y. Kita, S.R. Choudhury; KMIC: I. Suh, S.H. Jee, I.S. Kim; Melbourne: G.G. Giles; Miyama: T. Hashimoto, K. Sakata; Newcastle: A. Dobson; Ohasama: Y. Imai, T. Ohkubo, A. Hozawa; Perth: the late K. Jamrozik, M. Hobbs, R. Broadhurst; Saitama: K. Nakachi; Seven Cities: X.H. Fang, S.C. Li, Q.D. Yang; Shanghai Factory Workers: Z.M. Chen; Shibata: H. Tanaka; Shigaraki Town: Y. Kita, A. Nozaki, H. Ueshima; Shirakawa: H. Horibe, Y. Matsutani, M. Kagaya; Singapore Heart: K. Hughes, J. Lee; Singapore NHS92: D. Heng, S.K. Chew; Six Cohorts: B.F. Zhou, H.Y. Zhang; Tanno/Soubetsu: K. Shimamoto, S. Saitoh; Tianjin: Z.Z. Li, H.Y. Zhang; Western Australia AAA Screenees: P. Norman, the late K. Jamrozik; Xi’an: Y. He, T.H. Lam; Yunnan: S.X. Yao.

## Competing interests

The authors declare that they have no competing interests.

## Authors’ contributions

RH conceived the manuscript, interpreted the data and wrote the manuscript^;^ FB analyzed the data; JW, GG, THL, TO, SHJ, XF acquired the data and provided critical revision of the manuscript; KZ, SK provided critical revision of the manuscript and MW oversaw the statistical analysis and provided critical revision of the manuscript. All authors read and approved the final manuscript.

## Pre-publication history

The pre-publication history for this paper can be accessed here:

http://www.biomedcentral.com/1471-2261/14/61/prepub

## Supplementary Material

Additional file 1: WebFigure 1 Hazard ratios for fatal heart failure associated with a 10 mmHg increment in systolic blood pressure, adjusted for age, smoking, body mass index, diabetes, and regression dilution bias and stratified by sex and study in the Asia Pacific Cohort Studies Collaboration, both by subgroup (male versus female; Asia versus Australia; < 75 yrs vs> 75 yrs) and overall. Bars show 95% confidence intervals. The vertical dimension of the diamond indicates the overall estimate and the horizontal dimension indicates the 95% confidence interval. **WebFigure 2.** Hazard ratios for fatal heart failure associated with cigarette smoking (ever versus never) adjusted for age, systolic blood pressure, body mass index and stratified by sex and study in the Asia Pacific Cohort Studies Collaboration. Conventions as in **WebFigure 1.WebFigure 3.** Hazard ratios for fatal heart failure associated with diabetes (yes versus no) adjusted for age, systolic blood pressure, body mass index, cigarette smoking, regression dilution bias and stratified by sex and study in the Asia Pacific Cohort Studies Collaboration. Conventions as in **WebFigure 1.WebFigure 4.** Hazard ratios for fatal heart failure associated with 1 mmol/L increment in total cholesterol (multiply by 38.7 to obtain mg/dL) adjusted for age, systolic blood pressure, body mass index, cigarette smoking, regression dilution bias and stratified by sex and study in the Asia Pacific Cohort Studies Collaboration. Conventions as in **WebFigure 1.WebTable 1.** Associations between risk factors and mortality from heart failure in those with and without a history of cardiovascular disease at study baseline. Click here for file
