# Supplementary material for: A comparison of risk factors for mortality from heart failure in Asian and non-Asian populations: An overview of individual participant data from 32 prospective cohorts from the Asia-Pacific Region
Source: BMC Cardiovasc Disord. 2014 May 3;14:61. doi: 10.1186/1471-2261-14-61 (PMC4037783; doi:10.1186/1471-2261-14-61)
Supplement: Additional file 1: WebFigure 1 — Hazard ratios for fatal heart failure associated with a 10 mmHg increment in systolic blood pressure, adjusted for age, smoking, body mass index, diabetes, and regression dilution bias and stratified by sex and study in the Asia Pacific Cohort Studies Collaboration, both by subgroup (male versus female; Asia versus Australia; < 75 yrs vs> 75 yrs) and overall. Bars show 95% confidence intervals. The vertical dimension of the diamond indicates the overall estimate and the horizontal dimension indicates the 95% confidence interval. WebFigure 2. Hazard ratios for fatal heart failure associated with cigarette smoking (ever versus never) adjusted for age, systolic blood pressure, body mass index and stratified by sex and study in the Asia Pacific Cohort Studies Collaboration. Conventions as in WebFigure 1.WebFigure 3. Hazard ratios for fatal heart failure associated with diabetes (yes versus no) adjusted for age, systolic blood pressure, body mass index, cigarette smoking, regression dilution bias and stratified by sex and study in the Asia Pacific Cohort Studies Collaboration. Conventions as in WebFigure 1.WebFigure 4. Hazard ratios for fatal heart failure associated with 1 mmol/L increment in total cholesterol (multiply by 38.7 to obtain mg/dL) adjusted for age, systolic blood pressure, body mass index, cigarette smoking, regression dilution bias and stratified by sex and study in the Asia Pacific Cohort Studies Collaboration. Conventions as in WebFigure 1.WebTable 1. Associations between risk factors and mortality from heart failure in those with and without a history of cardiovascular disease at study baseline. [file 1471-2261-14-61-S1.docx]

**ADDITIONAL FILE**

**WebFigure 1** Hazard ratios for fatal heart failure associated with a 10mmHg increment in systolic blood pressure, adjusted for age, smoking, body mass index, diabetes, and regression dilution bias and stratified by sex and study in the Asia Pacific Cohort Studies Collaboration, both by subgroup (male versus female; Asia versus Australia; < 75 yrs vs> 75 yrs) and overall. Bars show 95% confidence intervals. The vertical dimension of the diamond indicates the overall estimate and the horizontal dimension indicates the 95% confidence interval.

**WebFigure 2** Hazard ratios for fatal heart failure associated with cigarette smoking (ever versus never) adjusted for age, systolic blood pressure, body mass index and stratified by sex and study in the Asia Pacific Cohort Studies Collaboration. Conventions as in Additional Figure 1.

**WebFigure 3** Hazard ratios for fatal heart failure associated with diabetes (yes versus no) adjusted for age, systolic blood pressure, body mass index, cigarette smoking, regression dilution bias and stratified by sex and study in the Asia Pacific Cohort Studies Collaboration. Conventions as in Additional Figure 1.

**WebFigure 4** Hazard ratios for fatal heart failure associated with 1 mmol/L increment in total cholesterol (multiply by 38.7 to obtain mg/dL) adjusted for age, systolic blood pressure, body mass index, cigarette smoking, regression dilution bias and stratified by sex and study in the Asia Pacific Cohort Studies Collaboration. Conventions as in Additional Figure 1.


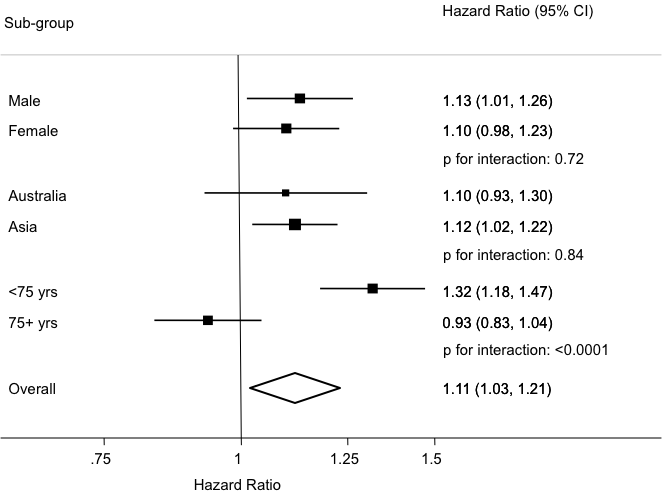
 WebFigure 1

WebFigure 2.


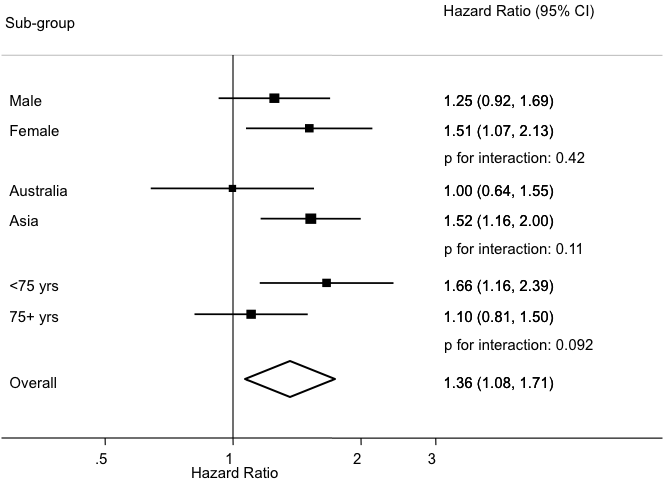


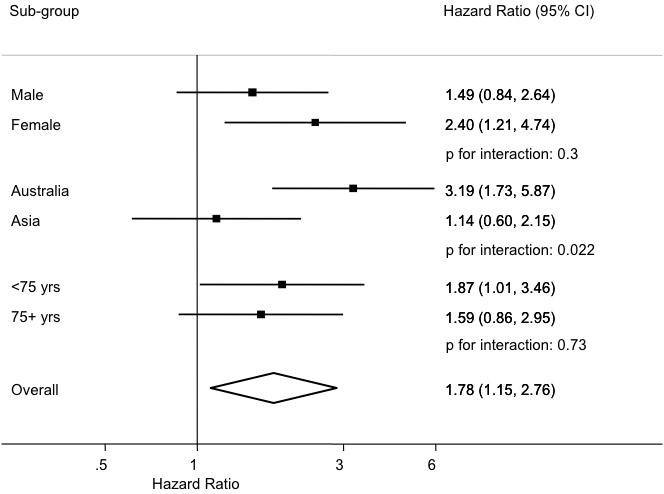
 WebFigure 3.


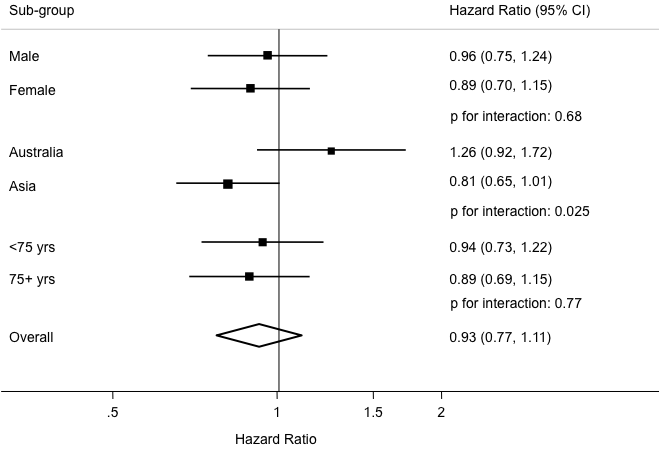
 WebFigure 4

WebTable 1

Associations between risk factors and mortality from heart failure in those with and without a history of cardiovascular disease at study baseline

| Risk factor | Number of participants | No. Heart failure deaths | Hazard Ratio (95% CI) | P for interaction |
| --- | --- | --- | --- | --- |
| 10mmHg SBP |  |  |  |  |
| No CVD history | 140151 | 393 | 1.13 (1.04-1.22) | 0.024 |
| CVD history | 12913 | 89 | 0.99 (0.85-1.16) |  |
| Body mass index |  |  |  |  |
| No CVD history | 137927 | 332 | 0.97 (0.94-1.00) | 0.92 |
| CVD history | 12350 | 63 | 0.97 (0.91-1.03) |  |
| Smoking (Ever vs never) |  |  |  |  |
| No CVD history | 139422 | 388 | 1.44 (1.12-1.85) | 0.10 |
| CVD history | 12882 | 87 | 1.33 (0.86-2.07) |  |
| Diabetes (Yes vs No) |  |  |  |  |
| No CVD history | 119757 | 354 | 1.85 (1.15-2.96) |  |
| CVD history | 11802 | 81 | 3.27 (1.71-6.27) | 0.15 |
| Total cholesterol 1mmol/L |  |  |  |  |
| No CVD history | 101352 | 238 | 0.86 (0.70-1.06) | 0.45 |
| CVD history | 6409 | 41 | 1.02 (0.68-1.53) |  |
